# RIG-I Promotes Tumorigenesis and Confers Radioresistance of Esophageal Squamous Cell Carcinoma by Regulating DUSP6

**DOI:** 10.3390/ijms24065586

**Published:** 2023-03-15

**Authors:** Lu Li, Lei Lv, Jun-Chao Xu, Qing He, Na Chang, Ya-Yun Cui, Zhen-Chao Tao, Tao Zhu, Li-Ting Qian

**Affiliations:** 1 Department of Oncology Radiotherapy, The First Affiliated Hospital of USTC, Division of Life Sciences and Medicine, University of Science and Technology of China, Hefei 230031, China; lilu2018@mail.ustc.edu.cn (L.L.);; 2 The CAS Key Laboratory of Innate Immunity and Chronic Disease, Division of Life Sciences and Medicine, University of Science and Technology of China, Hefei 230027, China

**Keywords:** RIG-I, esophageal squamous cell carcinoma, radiosensitivity, G2/M DNA damage checkpoint, tumor growth

## Abstract

We investigated the expression and biological function of retinoic acid inducible gene I (RIG-I) in esophageal squamous cell carcinoma (ESCC). Materials and methods: An immunohistochemical analysis was performed on 86 pairs of tumor tissue and adjacent normal tissue samples of patients with ESCC. We generated RIG-I-overexpressing ESCC cell lines KYSE70 and KYSE450, and RIG-I- knockdown cell lines KYSE150 and KYSE510. Cell viability, migration and invasion, radioresistance, DNA damage, and cell cycle were evaluated using CCK-8, wound-healing and transwell assay, colony formation, immunofluorescence, and flow cytometry and Western blotting, respectively. RNA sequencing was performed to determine the differential gene expression between controls and RIG-I knockdown. Tumor growth and radioresistance were assessed in nude mice using xenograft models. RIG-I expression was higher in ESCC tissues compared with that in matched non-tumor tissues. RIG-I overexpressing cells had a higher proliferation rate than RIG-I knockdown cells. Moreover, the knockdown of RIG-I slowed migration and invasion rates, whereas the overexpression of RIG-I accelerated migration and invasion rates. RIG-I overexpression induced radioresistance and G2/M phase arrest and reduced DNA damage after exposure to ionizing radiations compared with controls; however, it silenced the RIG-I enhanced radiosensitivity and DNA damage, and reduced the G2/M phase arrest. RNA sequencing revealed that the downstream genes DUSP6 and RIG-I had the same biological function; silencing DUSP6 can reduce the radioresistance caused by the overexpression of RIG-I. RIG-I knockdown depleted tumor growth in vivo, and radiation exposure effectively delayed the growth of xenograft tumors compared with the control group. RIG-I enhances the progression and radioresistance of ESCC; therefore, it may be a new potential target for ESCC-targeted therapy.

## 1. Introduction

Esophageal cancer is the eighth most prevalent cancer and is the sixth leading cause of cancer-related deaths worldwide [1,2]. Adenocarcinoma and squamous cell carcinoma are two main histopathological subtypes of esophageal cancer. Esophageal squamous cell carcinoma (ESCC) accounts for >90% of the cases of esophageal cancer; its high prevalence has been reported mainly from East Asia to Central Asia [3,4]. ESCC has a poor prognosis, high mortality, and >15% 5-year survival rate [3,5]. Recent developments in early screening and multimodality therapeutic strategies have improved the clinical management and the overall survival of patients with ESCC [6,7]. Currently, neoadjuvant chemoradiotherapy is used to treat locally advanced patients [8]. Although radiotherapy (RT) is one of the most effective treatments for ESCC [9], radiotherapy resistance is a major reason for the poor survival and treatment failure in patients [10]. Therefore, improving radiosensitivity is essential for the radiation treatment of patients with ESCC. 

Retinoic acid inducible gene I (RIG-I) is a cytoplasmic pattern recognition receptor that produces an innate antiviral immune response by sensing viral RNA [11]. In addition, it has diverse functions as a pattern recognition receptor [12,13]. RIG-I plays a key role in immunotherapy [14,15]; the activation of RIG-I by the targeted nanoparticle delivery of agonists is the key to generating an immune response, thereby inducing tumor cell killing and enhancing the therapeutic effect [16,17,18,19]. Although the regulatory role in tumors has been demonstrated, the low expression of RIG-I in melanoma and colon cancer is associated with a poor prognosis [20,21]. RIG-I is overexpressed in ovarian, colorectal, and endometrial cancers; it plays an essential role in proliferation, apoptosis, and immune escape [22,23,24]. Therefore, RIG-I is a potential target for cancer treatment. RIG-I is involved in the regulation of radiotherapy by disrupting the synthesis of DNA repair proteins [25]. In breast cancer, RIG-I recognizes the exosome-derived RNA and confers radiochemotherapy resistance [26,27]. Taken together, RIG-I plays an important role in the radiotherapy-induced cell death and tumor suppression.

Dual-specificity phosphatase 6 (DUSP6) is a bispecific protein phosphatase subfamily member located in the cytoplasm, which regulates cell proliferation and differentiation by negatively regulating MAP kinase superfamily members [28]. Previous studies have demonstrated that DUSP6 is associated with many types of cancer and plays different roles; for example, DUSP6 regulates tumor suppression through ERK1/2 negative feedback in pancreatic cancer, lung cancer, ovarian cancer and nasopharyngeal carcinoma; on the contrary, DUSP6 plays an oncogenic role in human glioblastoma, thyroid cancer, breast cancer, and acute myeloid leukemia [29]. Moreover, some previous studies have demonstrated that the depletion of DUSP6 activates ATM-CHECK2 pathway proteins and delays the DNA damage repair [30], and the inhibition of the DUSP6 catalytic activity can lead to radiosensitization in glioblastoma [31]. In short, DUSP6 affects and determines the carcinogenic fate of specific cancers, and is involved in the DNA damage response and regulation of radiosensitivity.

In addition, a previous study found that TAF-derived exosomes promoted the proliferation and chemoresistance of ESCC through the RIG-I/IFN-β axis [32]. Nevertheless, the role of RIG-I in the progression and radioresistance of ESCC is still unclear. Therefore, we investigated the biological function of RIG-I in ESCC and evaluated its role in radioresistance. 

## 2. Results

### 2.1. High Expression of RIG-I in Human ESCC Tissue Samples and Cell Lines

**Figure 1:**
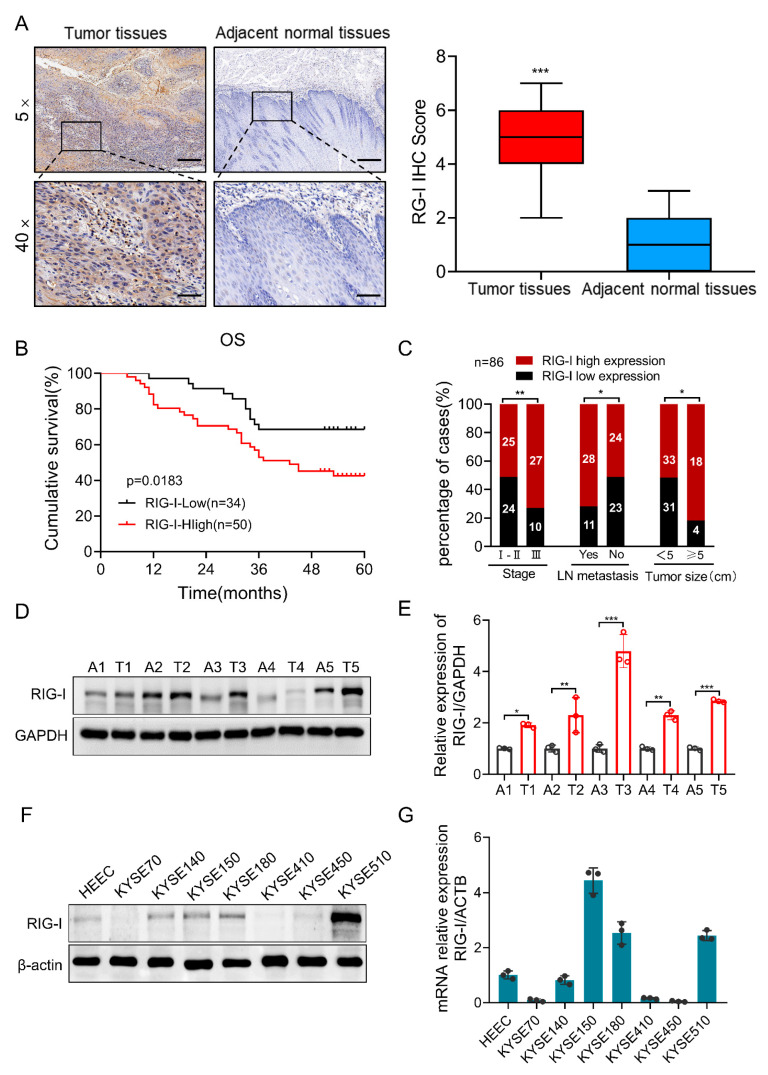
Protein and mRNA expression of RIG-I in ESCC tissues and adjacent normal tissues and cell lines. (**A**) The expression level of RIG-I protein in ESCC tissues and adjacent normal tissues was analyzed using immunohistochemistry (magnification 5× or 40×), Representative pictures are displayed; scale bar = 50 µm. Box plot showing immunohistochemical staining scores of RIG-I in ESCC samples (ESCC tissues = 86 and adjacent normal tissues = 86) (**B**) Kaplan–Meier analysis of the relationship between RIG-I protein expression and overall survival of patients with ESCC. (**C**) Correlation between RIG-I protein levels and clinicopathological parameters of patients with ESCC. (**D**) Protein and (**E**) mRNA expression levels of RIG-I in fresh ESCC cancer tissues and adjacent normal tissues were examined using Western blotting and qRT-PCR. GAPDH was used as an internal control. Expression of RIG-I (**F**) protein level and (**G**) mRNA level in HEEC and ESCC cells was detected using Western blotting and qRT-PCR. The mRNA values of three independent experiments were shown black dot. ACTB was used as an internal control. Data are shown as mean ± standard deviation (SD) from three independent experiments. * *p* < 0.05, ** *p* < 0.01, and *** *p* < 0.001.

We detected the expression of RIG-I in ESCC cancer tissues and adjacent normal tissues using immunohistochemistry. The results demonstrated that the RIG-I expression in cancer tissues was significantly higher than that in adjacent normal tissues (Figure 1A). In addition, the high RIG-I expression was associated with a shorter overall survival (OS) in patients with ESCC (Figure 1B). Further, we performed univariate and multivariate analyses and found that the high RIG-I expression was associated with an advanced tumor stage and lymph node metastasis (Figure 1C), but not with gender, age, differentiation, nerve invasion, and ki67 index (Supplementary Table S1). In addition, we have used the longest diameter of 5cm as the cutoff value of tumor size [33]. Notably, the high expression of RIG-I was correlated with the large tumor size of ESCC (Figure 1C). On the other hand, RIG-I protein and mRNA expression levels were detected in five pairs of fresh ESCC cancer and adjacent normal tissues using Western blotting and qRT-PCR, respectively. We found that the expression of RIG-I was increased in cancer tissues compared to that in adjacent normal tissues (Figure 1D,E). Similarly, the expression of RIG-I in six ESCC cell lines (KYSE70, KYSE140, KTSE150, KYSE180, KYSE410, KYSE450, and KYSE510) and HEEC was detected. The results demonstrated that RIG-I was upregulated in ESCC cells, especially in KYSE510 and KYSE150, but was slightly lower in KYSE70 and KYSE450, which were selected for further experiments (Figure 1F,G). These results indicated that RIG-I promotes the growth of ESCC tumors.

### 2.2. RIG-I Enhances Proliferation, Migration, and Invasion in ESCC Cells

**Figure 2:**
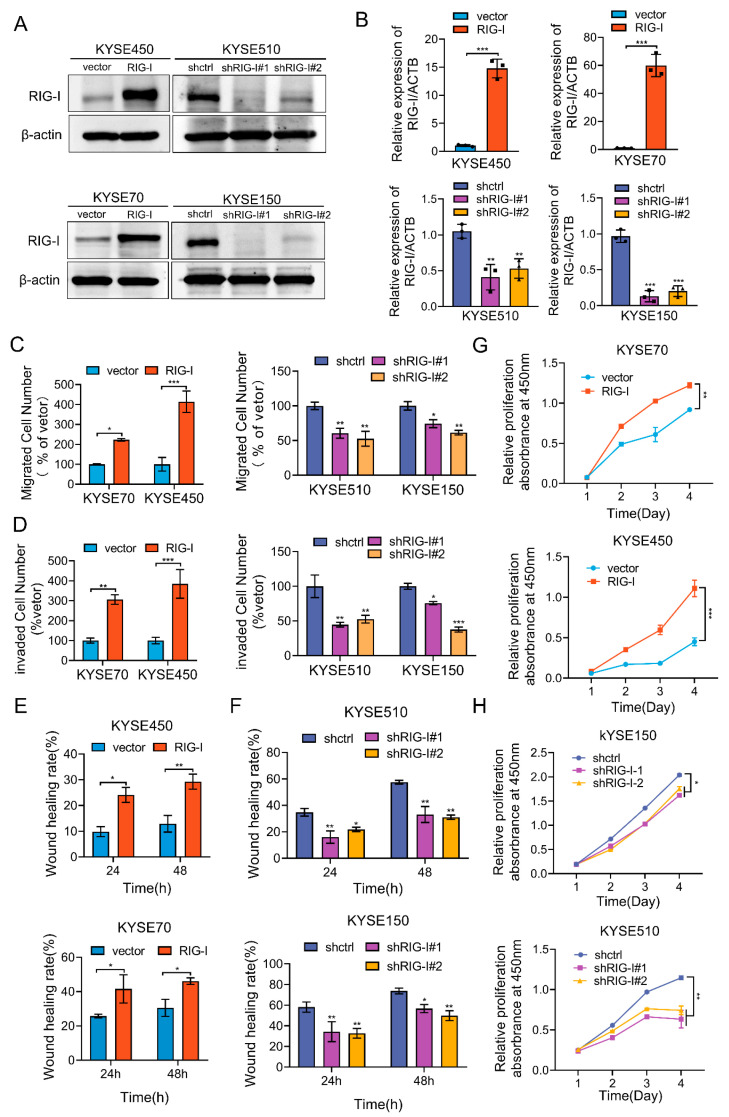
Effects of RIG-I overexpression (KYSE150 and KYSE510) and silencing (KYSE70 and KYSE450) on proliferation, migration, and invasion of ESCC cells. (**A**) RIG-I protein expression was detected using Western blotting and (**B**) mRNA level was detected using qRT-PCR. The mRNA values of three independent experiments were shown black dot, triangle and block. ACTB was used as an internal control. (**C**) Migration of ESCC cells overexpressing and lacking RIG-I was determined using a Matrigel-free transwell assay. (**D**) Invasion of ESCC cells overexpressing and lacking RIG-I was determined using Matrigel-containing transwell assay. Cell migration was detected using wound-healing assay (**E**) in RIG-I-overexpressing KYSE70 and KYSE450 cells (**F**) in RIG-I-lacking KYSE150 and KYSE510 cells. The CCK8 assay was used to determine the proliferation of (**G**) RIG-I-overexpressing cells and (**H**) RIG-I-knockdown cells. Empty vector and shRNA-control were used as controls. Data are shown as mean ± SD from three independent experiments. * *p* < 0.05, ** *p* < 0.01, and *** *p* < 0.001.

We established RIG-I overexpressing cell lines KYSE70 and KYSE450 and RIG-I knockdown cell lines KYSE150 and KYSE510 to investigate the biological function of RIG-I in ESCC. The results of Western blotting (Figure 2A) and qRT-PCR (Figure 2B) confirmed the RIG-I overexpression and knockdown in the respective cell lines. In the migration assay, the RIG-I-overexpressing KYSE70 and KYSE450 cells had a faster migration rate, whereas the RIG-I knockdown KYSE150 and KYS510 cells had a slower migration rate compared with the empty vector (Figure 2C, Supplementary Figure S1A). In the invasion assay, the invasion ability was significantly increased in high RIG-I-expressing and decreased in low RIG-I- expressing cell lines compared with the control group (Figure 2D and Supplementary Figure S1B). The variations in migration rate were also observed in the wound-healing assay; wound healing was faster in RIG-I-overexpressing cells and slower in RIG-I-knockdown cells compared with the empty vector and control (Figure 2E,F, Supplementary Figure S1C,D).

The CCK-8 assay demonstrated that the overexpression of RIG-I enhanced the proliferation of KYSE70 and KYSE450 cells (Figure 2G), whereas the knockdown of RIG-I inhibited the cell proliferation in KYSE150 and KYSE510 cells (Figure 2H).

### 2.3. RIG-I Confers Radioresistance to ESCC Cells

**Figure 3:**
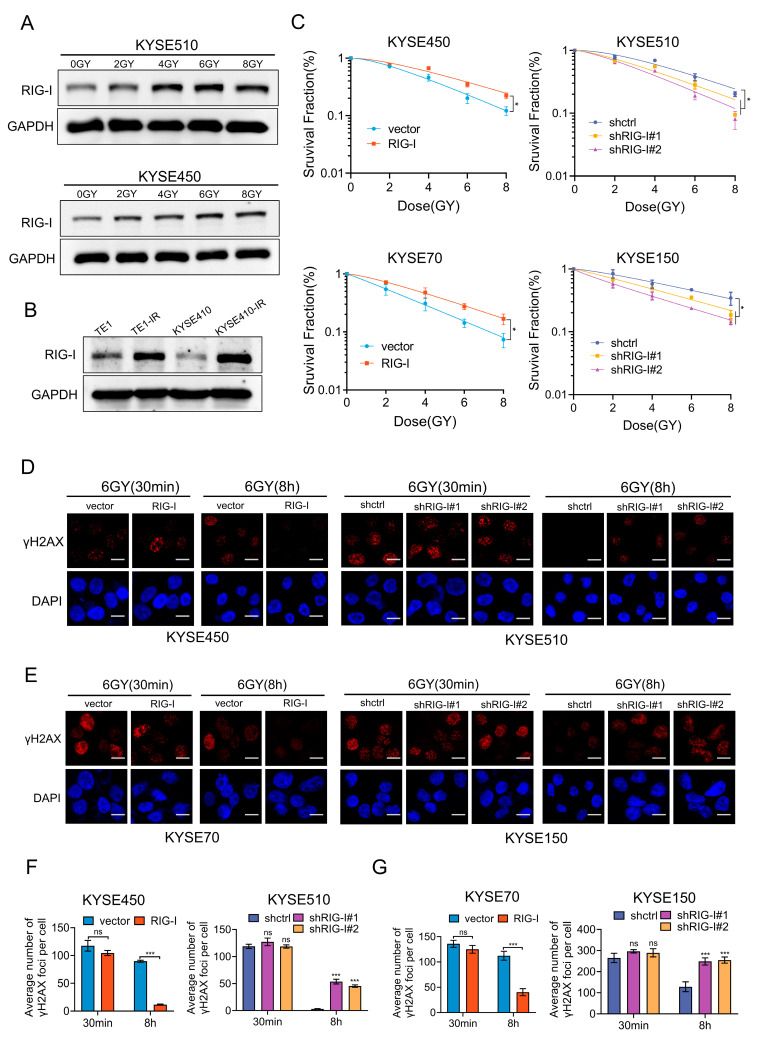
Effect of RIG-I overexpression and silencing on radioresistance of ESCC cells. (**A**) KYSE450 and KYSE510 were exposed to different doses of ionizing radiations (0, 2, 4, 6, and 8 Gy, 2 Gy/min), and RIG-I protein expression levels were detected using Western blotting. GAPDH was used as an internal control. (**B**) Protein levels of RIG-I in radiation-resistant TE-1 and KYSE410 cells and the corresponding naked cells (determined using Western blotting). GAPDH was used as an internal control. After stable RIG-I overexpression or depletion, (**C**) KYSE70, KYSE450, KYSE150, and KYSE510 cells were exposed to different doses (0, 2, 4, 6, and 8 Gy, 2 Gy/min) and cultured for 14 days to detect radiosensitivity using colony formation assay; cell survival curve was fitted by the single-hit multi-target model. (**D**,**E**) γ-H2AX foci levels in KYSE70, KYSE450, KYSE150, and KYSE510 cells were detected using immunofluorescence after 30 min and 8 h of 6Gy irradiation (40× magnification). Scale bar = 30 µm. Empty vector and shRNA-control as controls. (**F**,**G**) Bar graph represents the average number of γ-H2AX foci per cell in KYSE450, KYSE510, KYSE70, and KYSE150 cells. Empty vector and shRNA-control were used as controls. Data are shown as mean ± SD from three independent experiments, ns: not significant, * *p* < 0.05 and *** *p* < 0.001.

Radioresistance is a major reason for treatment failure in patients with ESCC [9,10]. Therefore, we investigated the association of elevated RIG-I levels with radioresistance. The RIG-I expression was markedly increased in a dose-dependent manner after exposure to different radiation doses (0, 2, 4, 6, and 8 GY) in KYSE450 and KYSE510 cells (Figure 3A). Moreover, the expression of RIG-I was higher in TE-1-R and KYSE410-R cell lines with permanent radioresistance than in control (radiosensitive) cell lines (Figure 3B). Colony formation assays demonstrated that the high expression of RIG-I led to radioresistance, whereas silencing RIG-I increased the radiosensitivity of ESCC cells compared to the control group (Figure 3C, Supplementary Figure S2A,B). The Sensitization Enhancement Ratios (SER) of the RIG-I group in KYSE70 and KYSR450 cells were 0.81 and 0.73, respectively (Supplementary Table S2), and the SER of shRIG-I#1 and shRIG-I#2 groups in KYSE150 and KYSE510 cells were 1.19, 1.26, 1.05, and 1.20, respectively (Supplementary Table S3). These results effectively demonstrated the vital role of RIG-I in regulating the radiosensitivity of ESCC cells. 

DNA double-strand breaks can be directly induced by irradiation, and the radiosensitivity of tumor cells is strongly associated with DNA damage repair [34]. RIG-I is involved in DNA damage repair [26]; therefore, we further explored the role of RIG-I in radioresistance. We examined γ-H2AX foci levels at 0.5 and 8 h in ESCC cells after exposure to 6Gy ionizing radiation using immunofluorescence. The results demonstrated that the γ-H2AX signal was significantly reduced after 8 h of irradiation in RIG-I-overexpressing cells; however, the knockdown of RIG-I reduced the radiation-induced DNA damage repair (Figure 3D–G).

### 2.4. DUSP6 Enhances the Radioresistance of ESCC Cells

**Figure 4:**
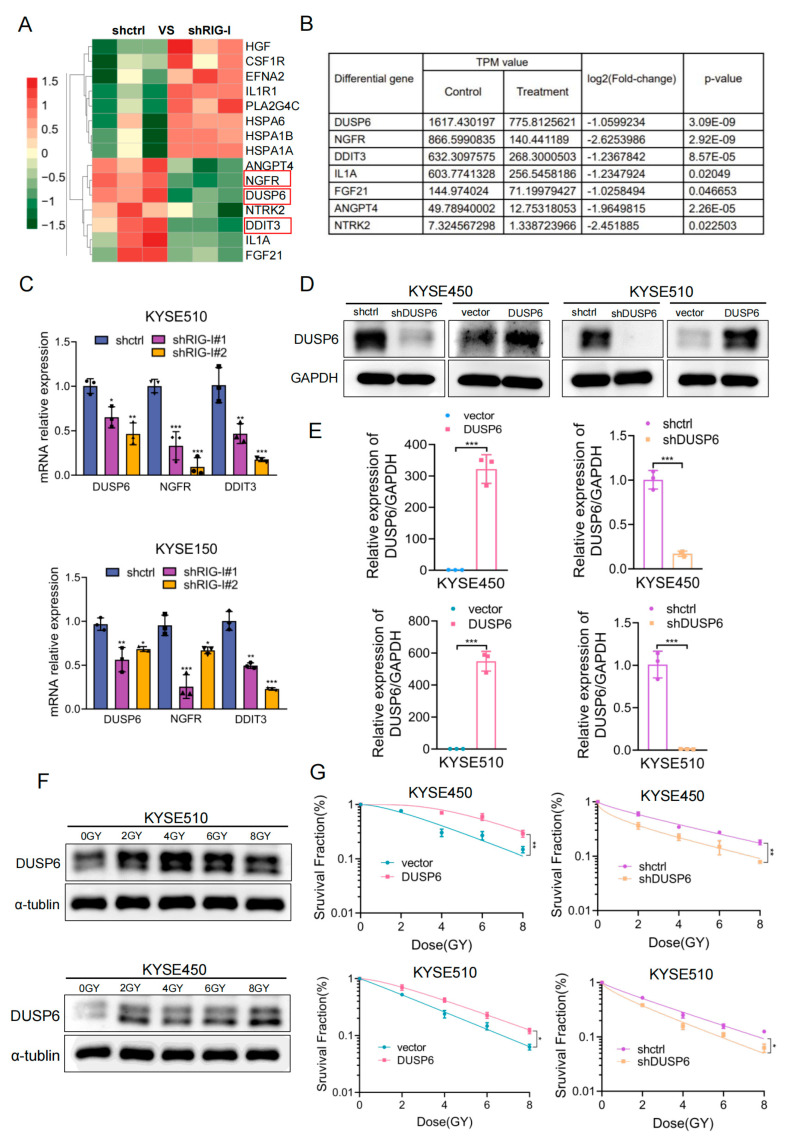
Effect of overexpression and silencing of downstream differential gene DUSP6 on radioresistance of ESCC cells. (**A**) Differentially expressed genes in the MAPK signaling pathway after RIG-I knockdown in KYSE510 cells were analyzed using RNA-seq. (**B**) TPM, log2, and *p* values of downregulated genes in the MAPK pathway were determined using RNA-seq analysis. shRNA-control was used as the control. (**C**) mRNA expression of screened DUSP6, NGFR, and DDIT3 genes in KYSE150 and KYSE510 cells was verified using RT-PCR. The mRNA values of three independent experiments were shown as dot, triangle or block. GAPDH was used as an inner control. Overexpression or knockdown of DUSP6 in KYSE450 and KYSE510 cells. (**D**) DUSP6 protein expression was quantified using Western blotting and (**E**) mRNA levels were quantified using qRT-PCR. Empty vector and shRNA-control were used as controls. The mRNA values of three independent experiments were shown as dot, triangle or block. GAPDH was used as an internal control. (**F**) KYSE450 and KYSE510 were exposed to different doses of ionizing radiations (0, 2, 4, 6, and 8 Gy, 2 Gy/min) and DUSP6 protein expression levels were quantified. GAPDH was used as an inner control. After stable DUSP6 overexpression or depletion using lentiviral vectors, (**G**) KYSE450 and KYSE510 cells were exposed to different doses (0, 2, 4, 6, and 8 Gy, 2 Gy/min) and cultured for 14 days to measure radiosensitivity using colony formation assay; cell survival curve fitted by the single-hit multi-target model. α-tubulin was used as an internal control. Empty vector and shRNA-control were used as controls. Data are shown as mean ± SD from three independent experiments. * *p* < 0.05, ** *p* < 0.01, and *** *p* < 0.001.

We attempted to elucidate the molecular mechanisms of the RIG-I-induced radioresistance in ESCC cells. For this purpose, KYSE510 cells stably transfected with RIG-I (shRIG-I and shctrl) were selected for RNA sequencing to screen potential targets of RIG-I. We identified 290 upregulated and 465 downregulated genes in shRNA-RIG-I cell lines compared with control cell lines (Supplementary Figure S2C). Notably, the KEGG pathway enrichment analysis revealed that RIG-I was the strongest correlated with the PI3K/Akt signaling pathway, but the TPM value of its differential genes was generally low; thus, we selected the MAPK signaling pathway that also had a strong correlation with RIG-I (Supplementary Figure S2D). All the differential genes in the MAPK signaling pathway were listed (Figure 4A), and the three genes with the highest TPM (Transcripts Per Kilobase of exon model per Million mapped reads) value, DUSP6, NGFR, and DDIT3, were selected for further studies (Figure 4B). The results of qRT-PCR demonstrated that the mRNA expression of DUSP6, NGFR, and DDIT3 was significantly decreased in RIG-I-knockdown KYSE510 and KYSE150 cell lines (Figure 4C). 

DUSP6 may have a potential role in radiotherapy because it regulates DNA damage responses [30], and inhibition of its catalytic activity leads to the radiosensitization of glioblastoma cells [31]. We detected the protein and mRNA expression of DUSP6, and the stable cell lines overexpressing or lacking DUSP6 were successfully established in KYSE450 and KYSE510, respectively (Figure 4D,E). DUSP6 expression increased in a dose-dependent manner in KYSE450 and KYSE510 cells (Figure 4F) following radiation exposure (0, 2, 4, 6, and 8 Gy). Cell survival fraction and cloning experiments demonstrated that DUSP6-overexpressing cells were radioresistant, whereas DUSP6-knockdown cells were radiosensitive (Figure 4G, Supplementary Figure S2E,F). The SER of shDUSP6 was 1.04 and 1.07 in KYSE450 and KYSE510, respectively (Supplementary Table S4), and the SER of DUSP6 was 0.97 and 0.98 in KYSE450 and KYSE510 (Supplementary Table S5). These results suggested that DUSP6 confers radioresistance to ESCC cells.

### 2.5. RIG-I Contributes to Radiation-Induced G2/M Phase Arrest in ESCC Cells

**Figure 5:**
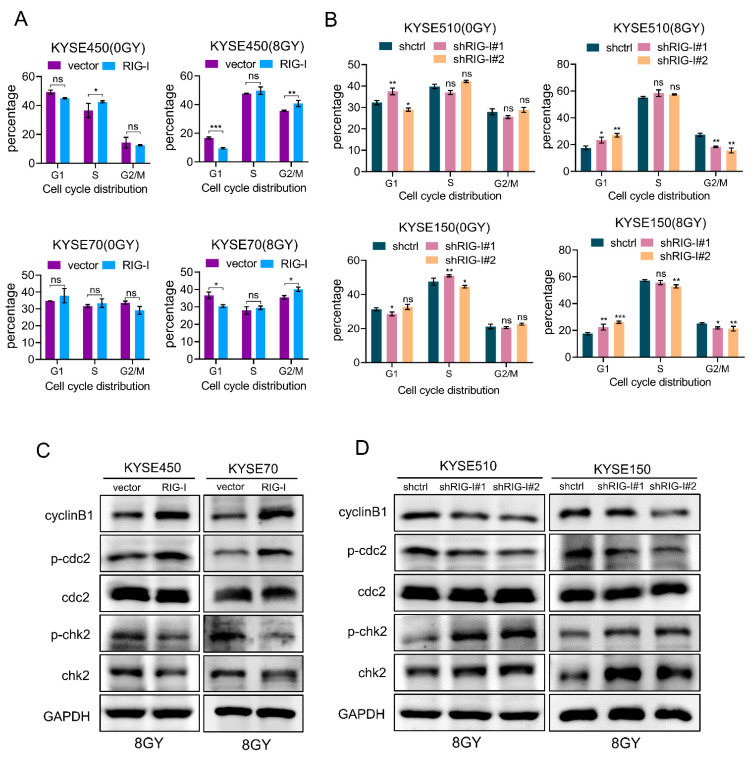
RIG-I causes radiation-induced G2/M phase arrest. (**A**) Cell cycle distribution upon overexpression of RIG-I in KYSE70 and KYSE450 cells with or without irradiation was assessed using flow cytometry. Empty vector was used as a control. (**B**) Cell cycle distribution upon knockdown of RIG-I in KYSE150 and KYSE510 cells with or without irradiation was assessed using flow cytometry. shRNA-control was used as a control. (**C**,**D**) G2/M phase DNA damage checkpoint signaling pathway-related proteins (detected using Western blotting) in RIG-I-overexpressing KYSE70 and KYSE450 cells and RIG-I-lacking KYSE150 and KYSE510 cells. Empty vector and shRNA-control were used as controls. GAPDH was used as an internal control. Data are shown as mean ± SD from three independent experiments. * *p* < 0.05, ** *p* < 0.01, and *** *p* < 0.001.

A major cause of radioresistance is cell cycle arrest, especially during the G2/M phase [35,36]. Therefore, to explore whether the RIG-I-induced ESCC radiotherapy resistance is related to cell cycle regulation, we detected the cell cycle distribution upon the overexpression or knockdown of RIG-I after ionizing the radiation exposure. The results demonstrated that a high expression of RIG-I induced the G2/M phase arrest after radiation exposure (Figure 5A, Supplementary Figure S3A,B), and the depletion of RIG-I significantly reduced the proportion of G2/M phase cells (Figure 5B, Supplementary Figure S3C,D) compared with the control group. The proportion of cell cycle distribution did not change significantly in the non-irradiated group. 

The G2/M phase DNA damage checkpoint is an important mechanism for cell cycle regulation [37]. Therefore, we detected the expression of cell cycle-related proteins and found that the expression of phospho-Chk2 and Chk2 was downregulated, and the cyclinB1 and phospho-cdc2 expression was enhanced compared with the control group in RIG-I-overexpressing cell lines after irradiation (Figure 5C). However, the down-regulation of RIG-I activated the expression of checkpoint kinase (Chk2) and phospho-Chk2, and decreased the expression of G2/M phase checkpoint protein cyclinB1 and phospho-cdc2 compared with the control group after irradiation (Figure 5D). Taken together, these results suggest that RIG-I promotes the radiation-induced cell cycle G2/M phase arrest. 

### 2.6. RIG-I-Induces Radioresistance and G2/M Phase Arrest by Regulating DUSP6 in ESCC Cells

**Figure 6:**
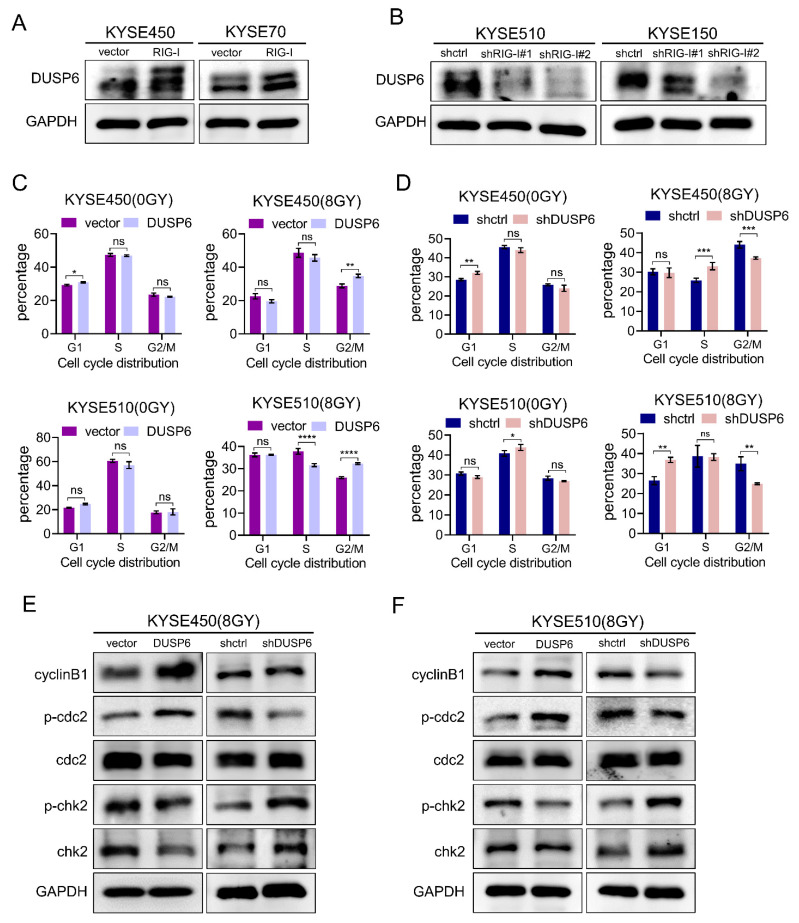
RIG-I targets downstream DUSP6 and causes radiation-induced G2/M arrest. Protein expression levels of DUSP6 were quantified using Western blotting (**A**) in RIG-I-overexpressing KYSE450 and KYSE70 cells (**B**) and RIG-I-lacking KYSE150 and KYSE510 cells. GAPDH was used as an internal control. Cell cycle distribution with or without irradiation was assessed in KYSE450 and KYSE510 (**C**) with overexpression of DUSP6 and (**D**) with knockdown of DUSP6 using flow cytometry. GAPDH was used as an internal control. G2/M phase DNA damage checkpoint signaling pathway-related proteins were detected in KYSE450 and KYSE510 cells (**E**) with overexpression of DUSP6 and (**F**) with knockdown of DUSP6 (using Western blotting). GAPDH was used as an internal control. Empty vector and shRNA-control were used as controls. Data are shown as mean ± SD from three independent experiments, ns: not significant, * *p* < 0.05, ** *p* < 0.01, *** *p* < 0.001, and **** *p* < 0.0001.

We first examined the protein expression level of DUSP6 in RIG-I lacking or overexpressing ESCC cells to confirm whether DUSP6 is involved in regulating the G2/M checkpoint. The results demonstrated that DUSP6 was significantly upregulated in RIG-I-overexpressing KYSE450 and KYSE70 cells (Figure 6A), whereas DUSP6 was downregulated in RIG-I-lacking KYSE510 and KYSE150 cells (Figure 6B). Next, we evaluated the cell cycle distribution of these cell lines using flow cytometry and found that the high expression of DUSP6 promoted the G2/M phase arrest after radiation exposure (Figure 6C, Supplementary Figure S4A,B). The G2/M phase arrest did not occur in DUSP6-knockdown cells (KYSE450 and KYSE510) compared with the control group (Figure 6D, Supplementary Figure S4C,D). There was no significant difference in the proportion of mitotic cells under non-irradiation conditions. 

In addition, we detected G2/M phase DNA damage checkpoint-related proteins. The results demonstrated that the expression of Chk2 and phospho-Chk2 was downregulated in DUSP6-overexpressing ESCC cells (KYSE450 and KYSE510), which promoted the expression of cyclinB1 and phospho-cdc2. However, the depletion of DUSP6 increased the expression of Chk2 and phospho-Chk2, and reduced the expression of cyclinB1 and phospho-cdc2 expression in ESCC cells (KYSE450 and KYSE510) (Figure 6E,F). 

**Figure 7:**
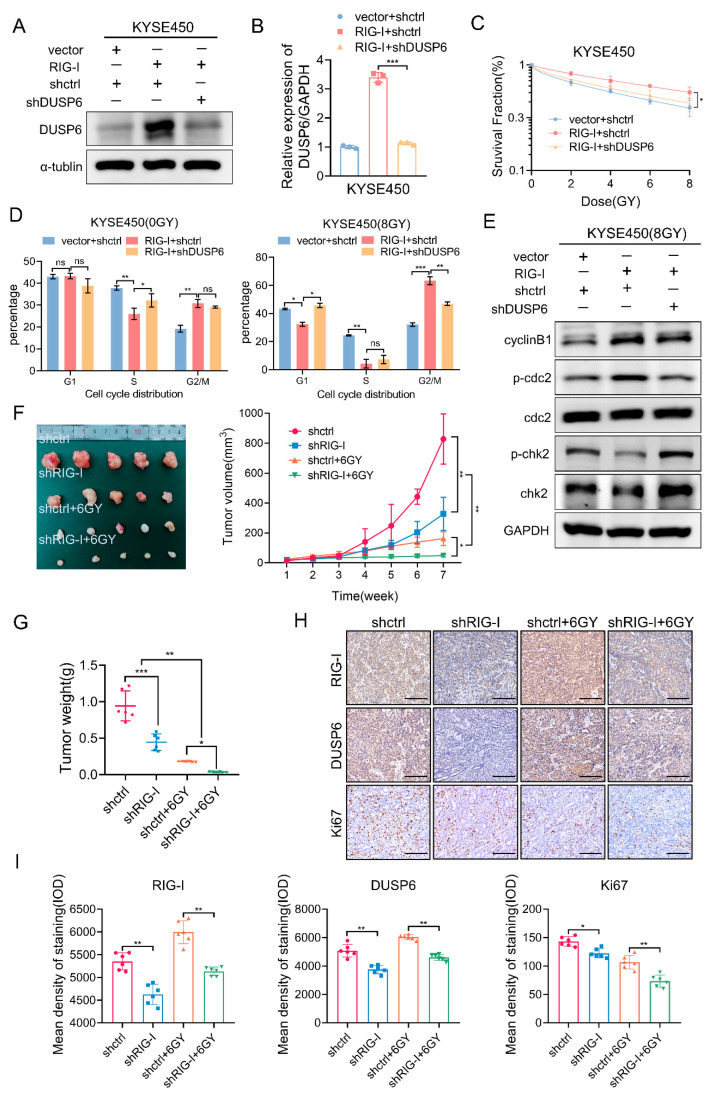
RIG-I enhances the radioresistance of ESCC cells by regulating DUSP6 in vivo or in vitro. Knockdown of DUSP6 in RIG-I overexpressing cells was successfully established. (**A**) DUSP6 protein levels were validated using Western blotting, and (**B**) DUSP6 mRNA levels were validated using qRT-PCR in KYSE450 cells. α-tubulin and GAPDH were used as internal controls. After lentivirus transduction of RIG-I overexpression and DUSP6 RNA interference stable transduction, (**C**) KYSE450 cells were exposed to different doses (0, 2, 4, 6, and 8 Gy, 2 Gy/min) and cultured for 14 days to measure radiosensitivity using colony formation assay; cell survival curve was fitted by the single-hit multi-target model. (**D**) Cell cycle distribution of KYSE450 cells with or without irradiation was assessed using flow cytometry. (**E**) G2/M phase DNA damage checkpoint signaling pathway-related proteins were detected in KYSE450 cells using Western blotting. GAPDH was used as an internal control. Empty vector + shRNA-control and vector-RIG-I + shRNA-control were used as controls. Data are shown as mean ± SD from three independent experiments. KYSE510 cells stably transfected with shRNA-RIG-I and shRNA-control were subcutaneously injected into the hind limbs of nude mice (n = 5 per group), and yshctrl + 6Gy and shRIG-I + 6Gy group were irradiated with 6Gy ionizing radiations 2 weeks after injection. (**F**) Transplanted tumors (tumor volume was measured every 7 days). (**G**) Tumor weight (g). The weight values of each tumor are displayed with dot, triangle, inverted triangle and block. (**H**,**I**) Immunohistochemical analysis of RIG-I, DUSP6, and Ki67 in RIG-I-silenced KYSE510 tumor-bearing nude mice with or without irradiation (40× magnification). Scale bar = 50 µm. The mean density of staining (IOD)was shown as dot, triangle, inverted triangle and block was on the histogram. Data are shown as mean ± SD, ns: not significant, * *p* < 0.05, ** *p* < 0.01, and *** *p* < 0.001.

These results indicate that DUSP6 is a downstream target gene of RIG-I leading to radiotherapy resistance and G2/M phase arrest in ESCC cells. We confirmed our results further. qRT-PCR and Western blotting results demonstrated that we successfully established the stable transfection of shRNA-DUSP6 and vector-RIG-I or shRNA-Control and vector-RIG-I, and vector-control and shRNA-control (Figure 7A,B). The cell survival fraction, determined using a clonogenic assay, demonstrated that silencing DUSP6 reduced the radioresistance of RIG-I-overexpressing ESCC cells (Figure 7C, Supplementary Figure S4E). The SER of the shRNA-control and vector-RIG-I group was 0.85 and the shRNA-DUSP6 and vector-RIG-I group was 1.12 (Supplementary Table S6). Further, we observed that the radiation-induced G2/M phase arrest caused by the RIG-I overexpression was eliminated. (Figure 7D, Supplementary Figure S4F). In addition, cell cycle-related proteins were detected. The results demonstrated that the depleted DUSP6 compensated for the decrease in Chk2 and phospho-Chk2 caused by the high expression of RIG-I, and the expression of the G2/M checkpoint protein cyclinB1 and phospho-cdc2 was lowered after irradiation (Figure 7E), which restored the radiosensitivity of ESCC cells.

### 2.7. RIG-I Promotes ESCC Tumorigenesis and Radioresistance In Vivo

We constructed a xenograft tumor model to demonstrate the effect of RIG-I on ESCC proliferation in vivo. The rate of tumor formation of RIG-I knockdown cells was significantly slower than that of shRNA-control cells, especially after irradiation (Figure 7F). Furthermore, tumors formed by RIG-I-depleted cells weighed less than those formed by empty shRNA-control cells after irradiation (Figure 7G). Immunohistochemical analysis showed that the RIG-I signal was weak, and the corresponding Ki67 signal and DUSP6 signal were also weak; irradiated tissues with low expression of RIG-I exhibited weaker Ki67 and DUSP6 signals, which was consistent with our in vitro results (Figure 7H,I).

## 3. Discussion

The comprehensive treatment of patients with ESCC usually includes chemotherapy, chemoradiotherapy, and surgical resection [1]; of these, radiotherapy is one of the most effective treatments [9]. However, radiation resistance is a major cause of poor survival and high recurrence in patients with ESCC [10]. Novel targeted therapies have been demonstrated to play an important role in the treatment of ESCC [38]. Therefore, the identification of potential molecular targets for targeted therapies can improve radiosensitivity, thereby increasing the clinical efficacy of the ESCC treatment.

RIG-I plays an important role in the immune response [17,19] and DNA damage repair [25,39]. Previous studies have found that RIG-I was activated by TAF-derived exosomes to up-regulate IFN/β, leading to cisplatin resistance in ESCC cells [32]. However, the role of RIG-I in ESCC has not been reported, and the role of RIG-I in ESCC radiation response is unclear. Therefore, we investigated the biological function of RIG-I in ESCC and found that the RIG-I expression was elevated in ESCC tissues compared to matched adjacent normal tissues and was associated with a poor prognosis. Moreover, the overexpression of RIG-I regulated the G2/M DNA damage checkpoints, thereby increasing the ESCC radioresistance by regulating DUSP6. Accordingly, RIG-I may be a new therapeutic target for increasing the radiosensitivity of ESCC tissues.

Patients with advanced ESCC often experience dysphagia due to the proliferation of malignant cells [1,3]. In addition, ESCC cell migration and invasion can lead to metastasis [1,3]. RIG-I plays an important regulatory role in the development of a variety of tumors [20,21,22,23,24,26]; its expression is upregulated in ovarian [22], colorectal [23], and endometrial cancers [24]. This study, for the first time, confirmed the carcinogenic effect of RIG-I in ESCC. The overexpression of RIG-I in ESCC cells promoted cell proliferation and increased cell migration and invasion, whereas the depletion of RIG-I inhibited cell proliferation and decreased cell migration and invasion. Previous evidence has demonstrated that the consumption of RIG-I inhibited cell viability and promoted apoptosis in colon cancer cells [23]; moreover, the overexpression of RIG-I promoted apoptosis and death in hepatocellular carcinoma [40]. The above suggested that RIG-I promotes ESCC cell proliferation; therefore, migration and invasion may be related to the inhibition of apoptosis.

RIG-I is involved in the DNA damage repair pathway [25,39]; specifically, the overexpressed RIG-I is recruited to repair radiation-induced DNA double-strand breaks, and it binds to repair proteins to inhibit DNA damage repair [25]. Furthermore, the deletion of the single-strand break repair protein induces an increase in RIG-I expression, which hinders DNA repair [39]. RIG-I plays a critical role in the radioresistance of breast cancer tissues, and the expression of RIG-I promotes the radiochemotherapy resistance of breast cancer [26,27]. Taken together, RIG-I may be involved in the regulation of radiotherapy. We determined that the high expression of RIG-I conferred radioresistance to the ESCC cells, whereas RIG-I silencing enhanced the radiosensitivity of ESCC cells. RIG-I plays an anticancer role in pancreatic [17] and colorectal cancers [23] through nanoparticle presentation or siRNA targeting; however, the role of RIG-I in ESCC has not been explored. Our results elaborated on the role of RIG-I in ESCC and provided a baseline to develop novel targeted therapy for ESCC. 

RNA sequencing analysis was performed to further explore the specific mechanism of RIG-I-induced ESCC radiation resistance, and the downstream target gene DUSP6 of RIG-I was obtained according to the differential gene KEGG pathway bubble diagram and TPM value analysis. DUSP6 protein and mRNA expression was significantly downregulated in RIG-I-silenced cells. DUSP6 is a member of the bispecific protein phosphatase subfamily [41], which regulates the cell proliferation and differentiation by negatively regulating the members of the MAPK superfamily [28]. The deletion of DUSP6 activates Chk2 in the DNA damage response pathway and increases the expression of the phosphorylated Chk2, H2AX, and ATM [36,42], suggesting that DUSP6 is an important regulator in the DNA damage response. Moreover, the inhibition of the catalytic activity of DUSP6 enhances the radiosensitivity of glioblastoma [31]. Therefore, DUSP6 may be involved in the regulation of radioresistance. This study clearly demonstrates that the upregulation of DUSP6 confers radioresistance to ESCC cells, whereas the downregulation of DUSP6 leads to the radiosensitization of ESCC cells. These results suggest that DUSP6 is involved in radioresistance. 

For patients with advanced ESCC having metastatic or unresectable disease, postoperative chemoradiotherapy or radiotherapy is recommended [1,5]; however, more than half of the patients relapse after radiotherapy [1]. This recurrence is associated with radiotherapy resistance, which is one of the leading causes of ESCC treatment failure and a low survival rate [10,42]. Radiation therapy induces DNA double-strand breaks in cancer cells, leading to genomic instability and cell cycle arrest [43]. Conventionally, cells in the cell cycle are redistributed after exposure to ionizing radiation, with cells in the G2/M phase being the most sensitive [44]. However, no study specifically examined whether the knockdown or overexpression of RIG-I affects cell cycle changes in ESCC after radiation treatment. In the present study, a high expression of RIG-I led to the radiation-induced cell cycle G2/M arrest, upregulation of cyclinB1 and phospho-cdc2, downregulation of Chk2 and phospho-Chk2 in the G2/M DNA damage checkpoint signaling pathway [45,46]. In contrast, the knockdown of RIG-I abolished the radiation-induced G2/M arrest, decreased cyclin B1 and phospho-cdc2 expression, and increased the Chk2 and phosphor-Chk2 expression. In addition, the overexpression or silencing of DUSP6 resulted in cell cycle G2/M arrest or elimination of arrest, respectively. Subsequent results demonstrated that the overexpression of RIG-I resulted in ESCC radioresistance and G2/M arrest, which were rescued by the depletion of DUSP6. Therefore, targeting RIG-I may be a new strategy to improve the radiosensitivity of ESCC by regulating the cell cycle G2/M arrest. In summary, RIG-I targets DUSP6 leading to radiotherapy resistance by inducing the cell cycle G2/M arrest in ESCC cells.

This study has some limitations. First, the limited number of clinical samples may influence the correlation between RIG-I and the clinicopathological parameters of patients. Second, this study did not explore how RIG-I affects the expression of DUSP6 at the molecular level. Therefore, further research is needed to elucidate the specific molecular mechanism by which RIG-I regulates DUSP6.

## 4. Materials and Methods

### 4.1. Collection of Tissue Specimens from Patients

Patients with ESCC (n = 86) who underwent surgery at the Western District of the First Affiliated Hospital of the University of Science and Technology of China from 2015 to 2017 were included in this study. We collected 5 fresh cancer tissues and 5 corresponding adjacent normal tissues from these patients. None of the patients received any treatment before surgery, including chemotherapy, radiotherapy, or adjuvant therapy. These tissues were microscopically examined by a senior pathologist in the Department of Pathology of the First Affiliated Hospital of USTC (Hefei, China). All the patients with ESCC were followed up with for 5 years, which included following up with their clinicopathologic parameters. The protocol of the study was approved by the Medical Research Ethics Committee of the First Affiliated Hospital of the University of Science and Technology of China (Approval No.: 2021KY-140), and all patients signed a written informed consent. 

### 4.2. Immunohistochemistry

Immunohistochemical staining was performed as previously described [47]. After dewaxing, the sections were stained with anti-RIG-I (PK5323S, Abmart, Shanghai, China), anti-dual specificity phosphatase 6 (DUSP6) (ab76310, Abcam, Cambridgeshire, ENG, UK), and anti-ki67 (ab16667, Abcam) primary antibodies. The immunohistochemistry slides were evaluated by two pathologists who were blinded to clinical information. The staining intensity scoring of cancer cells was scored as 0, negative (no staining); 1, weak (light yellow); 2, moderate (yellow), and 3, strong (brownish yellow). The score of the percentage of positive cells was defined as 0 (0–10%), 1 (11–25%), 2 (26–45%), 3 (46–75%), and 4 (75–100%). The total histological score was the sum of the above two scores [48]. The histological score of 5 was considered a cutoff value for a tumor with high expression, whereas a score of ≤4 indicated a tumor with low expression.

### 4.3. Cell Lines and Culture

Human ESCC cell lines (KYSE70, KYSE140, KYSE150, KYSE 180, KYSE 410, KYSE450, and KYSE510) and human normal esophageal epithelial cells (HEEC) were obtained from the Chinese Academy of Cell Resource Center, Shanghai, China; the human embryonic kidney 293 cell line (HEK293T) was obtained from the American Biological Standards Resource Center (Rockville, MD, USA). Cells were cultured in modified RPMI Medium (Hyclone, Cytiva, UT, USA) containing 10% fetal bovine serum (GIBCO, Invitrogen Inc., SA, Brazil), and 1% penicillin/streptomycin (Beyotime, Shanghai, China) at 37 °C in an incubator with 5% CO_2_.

### 4.4. Lentivirus Packaging and Transduction

Lentivirus packaging was performed as previously described [49]. The enveloped (pMD2G, 4 μg) and packaged (psPAX2, 7.3 μg) plasmids were mixed with various target plasmids (11.3 μg each): Plko.1-DDX58-shRNA-A12,-B1; Plko.1-shctrl; Psin-DUSP6-3Xflag; and Psin-3Xflag-NC were cotransfected into HEK293T cells. Packaged with CaCl_2_ transfection reagent, the mixture was incubated for 15 min and added to the cells cultured in the antibiotic-free DMEM medium. After 12 h incubation, the culture medium was changed; the virus particles were collected at 36 and 48 h and stored at −80 °C. The cell transduction was performed as previously described [50]. ESCC cells in the logarithmic growth phase were transduced with an appropriate amount of lentivirus for 24 h using 3–5 µg/mL polybrene staining reagent. Cells were grown in the complete growth medium and 2–6 µg/mL puromycin was used for screening. In this study, the DDX58 shRNA was purchased from the Sigma-Aldrich shRNA library (Mian Wu lab of USTC, Heifei, Anhui, China). Plasmids PHBLV-DDX58-3xflag-ZsGreen-puro, PHBLV-3xflag-ZsGreen-puro, PHBLV-DUSP 6-shRNA1-ZsGreen-BSD, and PHBLV-ZsGreen-BSD were procured from HanBio Therapeutics (Shanghai, China). 

### 4.5. Real-Time RT-PCR

The total RNA was extracted from cells and tissues using Trizol (Invitrogen, Carlsbad, CA, USA). The cDNA template was generated by reverse transcription according to the manufacturer’s instructions. TranScript^®^RT Reagent Kit (Transgen, Beijing, China) and TranScript^®^ Top/Tip Green qPCR SuperMix Kit (Transgen) were used. The mRNA primer sequences for all the detected genes were as follows: RIG-I: forward: 5′-CCCTGGACCCTACCTACATC-3′ and reverse: 5′-CCCAACTTTCAAT GGCTTCA-3′; DUSP6: forward: 5′-GAAATGGCGATCAGCAAGACG-3′ and reverse: 5′-CGACGACTCGTATAGCTCCTG-3′; GAPDH: forward: 5′-TGCACCAC CAACTGCTTAGC-3′ and reverse: 5′-GGCATGGACTGTGGTCATGAG-3′; ACTB: forward: 5′-GTGGCCGAGGACTTTGATTG-3′ and reverse: 5′-CCTGTAAC AACG CATCTCATATT-3′. The GAPDH and ACTB genes were used as internal references. PCR was performed using the MX3000P real-time PCR system (Agilent Technologies, Palo Alto, CA, USA). The total volume of the reaction mixture was 20 µL and the amplification conditions were 94 ° for 30 s, 94 ° for 15 s, 60 ° for 30 s, 95 ° for 15 s, 60 ° for 60 s, and 95 ° for 15 s. The Ct value was defined as the number of PCR cycles per transcript threshold, and the value of 2^ −∆∆Ct^ was calculated to represent the relative mRNA expression of the target gene.

### 4.6. Western Blotting

Western blotting was performed as described earlier [45] using rabbit anti-RIG-I, rabbit anti-phospho-cdc2(Tyr15), rabbit anti-phospho-Chk2(Thr68) (Cell Signaling Technology Inc., Boston, MA, USA), and rabbit anti-DUSP6 (Abcam) monoclonal antibodies. In addition, rabbit anti-cyclinB1 and rabbit anti-CDK1(cdc2), and rabbit anti-Chk2 (Proteintech Group Inc., Wuhan, China) polyclonal antibodies were also used. All primary antibodies were diluted at 1:1000. The same membrane was stripped and reprobed with mouse anti-GAPDH, anti-β-actin, and anti-α-tubulin monoclonal antibodies (Proteintech Group Inc.) as internal controls. 

### 4.7. Cell Counting Kit-8 Assay

Cell counting kit 8 (CCK8) assay detected the viability of ESCC cells in each group. The cells were seeded in 96-well plates at a density of 3000 cells/well, the CCK8 assay solution (Topscience Inc., Shanghai, China) was added at the same time point on the 1st, 2nd, 3rd, and 4th day, absorbance was measured at the wavelength of 450 nm using a microplate reader (SpectraMax iD5, Molecular Devices, Silicon Valley, CA, USA), and the cell viability curve was obtained.

### 4.8. Cell Migration and Invasion Assays

In the cell migration assay, the cells were spread into the chamber plates (24-well format, Corning, NY, USA) without Matrigel (Corning), and the migrated cells were counted after 24 h. For the cell invasion assay, cells were plated into Matrigel-coated chambers and invasive cells were counted after 48 h. The cells were seeded at a density of 1 × 10^5^ cells/well.

### 4.9. Wound-Healing Assay

The cell migration rate detected using wound-healing assay. The cells in treatment and control groups were cultured to 80–90% confluence and scratches were made using a sterile 10µL pipette tip. The cell wounds were washed twice with PBS and then cultured in a serum-free medium. The cell healing was observed at 0, 24, and 48 h using a microscope (Olympus Corp, Tokyo, Japan) and analyzed using ImageJ software (V1.8.0.112).

### 4.10. Colony Formation Assay

A clone formation assay was performed as previously described [51]. Cells were plated in 6-well plates at a density of 250, 500, 1000, 2000, and 4000 cells/well and then exposed to 0, 2, 4, 6, or 8 GY X-rays after 12 h of attachment. After culturing at 37 °C for 14 days, the cells were fixed in methanol for 20 min and stained with 0.1% crystal violet (Beyotime) for 30 min. Finally, the number of clones containing at least 50 cells was counted under a microscope (Olympus Corp), and the single-hit multi-target model was used to fit the cell survival curve.

### 4.11. Immunofluorescence Assay

We performed immunofluorescence assays using rabbit anti-γH2AX (Ser139) foci monoclonal antibody (Cell Signaling Technology Inc.) to detect the DNA damage and repair [33]. Laser confocal microscopy (ZEISS LSM710, Oberkohen, Battenburg, Germany) was used to detect the immunofluorescence using 594-anti-Rabbit IgG (Thermo Fisher Scientific, Shanghai, China) and DAPI fluorescent dye (Beyotime).

### 4.12. Flow Cytometry

Cell cycle analysis was performed using flow cytometry as previously described [45]. ESCC cells were treated with 8 GY X-rays, collected 24 h later, washed with ice-cold PBS, fixed in ice-cold 75% ethanol, and stored at −20 ℃ overnight. Next, the cells were washed twice with iced PBS and resuspended in a working solution containing 0.1% RNaseA (100 µg/mL, Beyotime), 0.2% TritonX-100, and 1% propidium iodide staining solution (50 µg/mL), and incubated on ice for 30 min in the dark. ESCC cells without any radiation treatment were processed as negative controls. The distribution of the cell cycle was evaluated using the Fortessa flow cytometer (BD Biosciences, Franklin Lakes, NJ, USA).

### 4.13. Xenograft Tumor Model

The xenograft tumor model was constructed as described earlier [52]. Male BALB/C nude mice (5 weeks old, 20–25 g weight) were provided by the Anhui Provincial Hospital Animal Center, China. Animal experiments were approved by the Animal Ethics Committee of the First Affiliated Hospital of the University of Science and Technology of China. Nude mice were randomly divided into four groups (n = 5 in each group), namely shctrl (shRNA-control stably transduced KYSE510 cells), shRIG-I (shRNA-RIG-I stably transduced KYSE510 cells), shctrl+6GY (shctrl and irradiation), and shRIG-I+6GY (shRIG-I and irradiation) The xenografts were established by subcutaneous injection of 0.1 mL KYSE510 cells (4 × 10^6^ cells/mL) into the proximal right and left hind limbs of the mouse. The size of the tumor was measured every 7 days using a vernier caliper. The tumor volume (in mm^3^) was calculated as (length × width^2^)/2. Approximately 20 days after the injection, mice in the radiotherapy group received 6 Gy X-ray irradiation. All mice were sacrificed at week 7, and the tumor tissues were removed and weighed.

### 4.14. Statistical Analysis

All data are expressed as the mean ± standard deviation of at least three independent experiments. Data were analyzed using SPSS version 24.0 (SPSS Inc., Chicago, IL, USA); the one-way analysis of variance (ANOVA), independent sample t-test, and χ^2^ test were performed. The survival probability and survival difference were obtained using the Kaplan–Meier method and log-rank test, respectively. *p* < 0.05 was considered statistically significant.

## 5. Conclusions

RIG-I promotes ESCC progression and radioresistance and may be a new potential target for ESCC-targeted therapy, especially when combined with radiotherapy. However, comprehensive clinical studies are required in the future to confirm the role of RIG-I in ESCC.

## Data Availability

The data that support the findings of this study are available from the corresponding author. Data may be available upon request to interested researchers. Please send data requests to Li-Ting Qian, MD. Department of Oncology Radiotherapy, The First Affiliated Hospital of USTC, Division of Life Sciences and Medicine, University of Science and Technology of China.

## Supplementary Materials

The supporting information can be downloaded at: https://www.mdpi.com/article/10.3390/ijms24065586/s1.
