# Supplementary material for: RIG-I Promotes Tumorigenesis and Confers Radioresistance of Esophageal Squamous Cell Carcinoma by Regulating DUSP6
Source: Int J Mol Sci. 2023 Mar 15;24(6):5586. doi: 10.3390/ijms24065586 (PMC10052926; doi:10.3390/ijms24065586)
Supplement: Supplementary file 1 [file ijms-24-05586-s001.zip › Description for supplementary materials.pdf]

Supplementary Figure legends

Supplementary Figure S1. Effects of overexpression and silencing of RIG-I on migration, invasion, and wound healing of ESCC cells. Representative images of (A) migration (B) invasion (C) wound healing assay are displayed. magnification 20×. Scale bar = 30 μm

Supplementary Figure S2. RIG-I promotes radioresistance in ESCC cells by targeting downstream DUSP6. (A-B) Clone formation assay was used to detect the role of RIG-I in ESCC radioresistance. Representative images are displayed. (C) Volcano plot shows representative differentially expressed genes after RIG-I knockdown detected using RNA-seq analysis. (D) KEGG pathway bubble map showing enrichment pathway statistics for differentially expressed genes detected using RNA-seq analysis (E-F) Clone formation assay was used to detect the role of DUSP6 in ESCC radioresistance. Representative images are displayed.

Supplementary Figure S3. RIG-I is involved in radiation-induced G2/M phase arrest. (A-B) Representative images of cell cycle detection after RIG-I overexpression in KYSE70 and KYSE450 cell lines. (C-D) Representative images of cell cycle detection after RIG-I silencing in KYSE150 and KYSE510 cell lines.

Supplementary Figure S4. RIG-I targets downstream DUSP6 and causes radiation-induced G2/M arrest. (A-B) Representative images of cell cycle detection for DUSP6 overexpression in KYSE450 and KYSE510 cell lines. (C-D) Representative images of DUSP6 depleted cell cycle detection in KYSE450 and KYSE510 cell lines. After lentivirus transduction of RIG-I overexpression and DUSP6 RNA interference stable transduction, (E) Role of DUSP6 in RIG-I-induced ESCC radioresistance was examined by colony formation assay. (F) Representative pictures of cell cycle detection of DUAP6 knockdown in RIG-I overexpressed KYSE450 cell line.

Supplementary Table legends

Supplementary Table S1. Correlation analyses between RIG-I protein expression and clinicopathologic variables of 86 patients with esophageal squamous cell carcinoma.

Supplementary Table S2. RIG-I upregulation impairs the radiosensitivity of ESCC cells

Supplementary Table S3. RIG-I silencing increases the radiosensitivity of ESCC cells

Supplementary Table S4. DUSP6 knockdown enhances the radiosensitivity of the ESCC cells

Supplementary Table S5. DUSP6 overexpression depletes the radiosensitivity of the ESCC cells

Supplementary Table S6. Radiosensitization effect of DUSP6 silencing on esophageal cancer cells with upregulated RIG-I
