# Supplementary material for: RIG-I Promotes Tumorigenesis and Confers Radioresistance of Esophageal Squamous Cell Carcinoma by Regulating DUSP6
Source: Int J Mol Sci. 2023 Mar 15;24(6):5586. doi: 10.3390/ijms24065586 (PMC10052926; doi:10.3390/ijms24065586)
Supplement: Supplementary file 1 [file ijms-24-05586-s001.zip › Supplementary Table S1.pdf]

Supplementary Table S1. Correlation analyses of RIG-I protein expression in relation to clinicopathologic variables of 86 patients with esophageal squamous cells carcinoma

Clinicopathologic Parameter	n (n=86)	RIG-I Low expression	RIG-I High expression	P Value
**Age(years)**				0.578
<65	30	11	19	
≥65	56	23	33	
**Sex**				0.655
Male	66	26	40	
Female	20	8	12	
**Lymph node metastasis**				**0.010**
+	39	11	28	
-	47	23	24	
**Vascular or nerve invasion**				0.401
+	16	7	9	
-	70	27	43	
**Stage**				**0.007**
I+ II	49	24	25	
III	37	10	27	
**Grade**				0.273
I-II	53	24	29	
III	33	10	23	
**Ki67 index**				0.549
≥45%	62	24	38	
<45%	24	11	13	
**Tumor size (cm)**				**0.013**
≥5	22	4	18	
<5	64	33	31	
