# Supplementary material for: RIG-I Promotes Tumorigenesis and Confers Radioresistance of Esophageal Squamous Cell Carcinoma by Regulating DUSP6
Source: Int J Mol Sci. 2023 Mar 15;24(6):5586. doi: 10.3390/ijms24065586 (PMC10052926; doi:10.3390/ijms24065586)
Supplement: Supplementary file 1 [file ijms-24-05586-s001.zip › Supplementary Table S2.pdf]

Supplementary Table S2. RIG-I upregulation impairs the radiosensitivity of ESCC cells

Group	D0	Dq	SF2	SER
KYSE450				
Vector	2.99	1.69	0.73	
RIG-I	4.09	2.02	0.83*	0.73
KYSE70				
Vector	3.06	1.20	0.55	
RIG-I	3.77	1.71	0.73*	0.81
