# Supplementary material for: RIG-I Promotes Tumorigenesis and Confers Radioresistance of Esophageal Squamous Cell Carcinoma by Regulating DUSP6
Source: Int J Mol Sci. 2023 Mar 15;24(6):5586. doi: 10.3390/ijms24065586 (PMC10052926; doi:10.3390/ijms24065586)
Supplement: Supplementary file 1 [file ijms-24-05586-s001.zip › Supplementary Table S3.pdf]

Supplementary Table S3. RIG-I silencing increases the radiosensitivity of ESCC cells

Group	D0	Dq	SF2	SER
KYSE510				
shctrl	3.80	2.10	0.85	
shRIG-I#1	3.62	1.72	0.73*	1.05
shRIG-I#2	3.16	1.57	0.68*	1.20
KYSE150				
shctrl	5.92	2.08	0.82	
shRIG-I#1	4.97	1.70	0.70*	1.19
shRIG-I#2	4.70	1.36	0.58*	1.26
