# Supplementary material for: RIG-I Promotes Tumorigenesis and Confers Radioresistance of Esophageal Squamous Cell Carcinoma by Regulating DUSP6
Source: Int J Mol Sci. 2023 Mar 15;24(6):5586. doi: 10.3390/ijms24065586 (PMC10052926; doi:10.3390/ijms24065586)
Supplement: Supplementary file 1 [file ijms-24-05586-s001.zip › Supplementary Table S4.pdf]

Supplementary Table S4. DUSP6 knockdown enhances the radiosensitivity of ESCC cells

Group	D0	Dq	SF2	SER
KYSE450				
shctrl	5.32	1.39	0.58	
shDUSP6	5.13	0.73	0.37*	1.04
KYSE510				
shctrl	3.62	1.10	0.51	
shDUSP6	3.38	0.67	0.37*	1.07
