# Supplementary material for: RIG-I Promotes Tumorigenesis and Confers Radioresistance of Esophageal Squamous Cell Carcinoma by Regulating DUSP6
Source: Int J Mol Sci. 2023 Mar 15;24(6):5586. doi: 10.3390/ijms24065586 (PMC10052926; doi:10.3390/ijms24065586)
Supplement: Supplementary file 1 [file ijms-24-05586-s001.zip › Supplementary Table S5.pdf]

Supplementary Table S5. DUSP6 overexpression depletes the radiosensitivity of ESCC cells

Group	D0	Dq	SF2	SER
KYSE450				
Vector	2.91	1.65	0.71	
DUSP6	2.99	2.75	0.98*	0.97
KYSE510				
Vector	2.87	1.11	0.52	
DUSP6	2.94	1.63	0.71*	0.98
