# Supplementary material for: RIG-I Promotes Tumorigenesis and Confers Radioresistance of Esophageal Squamous Cell Carcinoma by Regulating DUSP6
Source: Int J Mol Sci. 2023 Mar 15;24(6):5586. doi: 10.3390/ijms24065586 (PMC10052926; doi:10.3390/ijms24065586)
Supplement: Supplementary file 1 [file ijms-24-05586-s001.zip › Supplementary Table S6.pdf]

Supplementary Table S6. Radiosensitization effect of DUSP6 silencing on esophageal cancer cells with RIG-I upregulated

Group	D0	Dq	SF2	SER
KYSE450				
vector+shctrl	15.26	2.13	0.68	
RIG-I+shctrl	17.94	2.64	0.83*	0.85
RIG-I+shDUSP6	16.05	2.28	0.73*	1.12
